# Research on the influence of payment methods on the control of medical insurance expenses—Based on empirical analysis of double difference

**DOI:** 10.3389/fpubh.2022.938831

**Published:** 2022-12-21

**Authors:** Juan Luo, Shuxin Wang, Lulu Dan, Rui Zhang, Liang Bi

**Affiliations:** School of Management, Shanghai University of Engineering Science, Shanghai, China

**Keywords:** medical insurance, fund, payment method, cost control, DID

## Abstract

**Background:**

The medical insurance system is constantly reformed and optimized. In order to control the cost of medical insurance funds, the medical insurance payment method has been reformed. The reform of the payment method can effectively control the medical insurance expenses.

**Method:**

In this paper, the annual data of 27 provinces from 2013 to 2017 were selected, and the cost control effect of the dual difference (DID) model of medical insurance payment method was analyzed.

**Results:**

The study found that the effect of the pilot reform of medical insurance payment mode was in line with the policy objectives and achieved the effect of cost control to a certain extent.

**Conclusion:**

The failure to significantly reduce the growth rate of the expenditure of medical insurance funds is not ideal to curb the excessive growth of health insurance funds. Therefore, strengthening the control of medical expenses, improving the control of medical insurance fund fees through the reform of payment methods are the effective ways to strengthen the control of medical insurance funds.

## Introduction

In recent years, the coverage of China's medical insurance system has gradually expanded, the level of security has been further improved, and the high pressure of medical insurance fund growth has forced a new round of medical insurance reform. With the introduction of various medical insurance reform policies in China, various provinces and cities have started to control medical insurance expenses from the level of medical insurance payment, thus reducing the payment pressure of medical insurance fund. However, the control of medical insurance costs can not be achieved overnight, which requires concerted action of stakeholders' communities. However, stakeholders' insured persons are in a relatively passive position relative to hospitals and medical insurance institutions. The control of medical insurance funds focuses on reducing the moral hazard of insured persons, and the interaction effect between medical insurance and medical institutions is more obvious. Specifically, medical insurance institutions hope that medical insurance funds can improve the efficiency of fund use and reduce the pressure of medical insurance funds, while medical institutions hope to get enough subsidies to improve the service capacity and operation of medical projects. Therefore, the reform of medical insurance payment method can be used to control the expenses.

Scholars' research on medical insurance cost control mainly focuses on using more appropriate payment methods to restrain the unreasonable growth of funds.

Foreign scholars study the influence of different payment methods on medical expenses Pauly (1) found the fact that the payment of medical insurance depends on the total cost charged by the medical service provider, optimizing the payment method of medical insurance is helpful to control the medical expenses. And Babiarz (2) through studying the relationship between performance-based payment and medical expenses, it is found that if hospitals implement performance-based payment, medical quality can be further improved in a short time, thus achieving the effect of reducing medical expenses. As for empirical research, Moreno-Serra and Wagstaff (3) studied the reform of medical insurance payment methods in 28 Eurasian countries from 1990 to 2004, and found that if hospitals implemented the payment according to diseases, compared with the payment according to medical items, the medical expenses would increase, but the medical quality and efficiency would be further improved. Yip and Eggleston (4) studied the reform of basic medical insurance payment mode in Hainan Province in 1997 by using the double difference method, and found that different payment methods adopted by hospitals were different. The prepayment system obviously reduced the medical expenses of hospitals, especially the two highest medical expenses-expensive drugs and high-tech medical treatment items.

Domestic researchers mainly focus on the field of medical insurance payment reform and medical insurance cost control. First of all, some researchers focus on the research of DRGs payment method reform: In view of the unreasonable growth of current medical insurance fund, Chang et al. (5) analyzed the main objectives of current medical insurance policy reform in China, and he introduced the payment methods of medical insurance in various countries. Tao et al. (6) found that the reform areas generally showed the trend of slow growth of medical insurance fund expenditure, slow growth of hospitalization expenses, lower proportion of drug consumption, lower average hospitalization days and lower per capita out-of-pocket proportion. Therefore, DRG mode showed the “positive effect” of fee control for doctors, medical insurance and patients. Li et al. (7) compared the changes of hospital technical efficiency and expenses before and after the reform of DRGs payment mode. By applying DEA calculation to the input-output data of 45 sample hospitals in 2017 and 2018, it was found that after the reform of DRGs, hospital technical efficiency did not improve and expenses did not decrease in the short term. Secondly, in recent years, there have been more and more researches on the effective fee control of the total prepayment system: From the micro-data of Guangdong Province, Qu et al. (8) found that this payment method has no obvious effect on reducing the hospitalization expenses of patients, but has significant effect on medical insurance payment. Li and Chu (9) studied the changes before and after the reform of medical insurance policy, and analyzed that the total prepayment system can reduce the payment level of medical insurance fund to a certain extent. Lang et al. (10) and Zhou et al. (11) found that after the total prepayment system was adopted, the growth rate of residents' medical expenses decreased significantly. Finally, on the basis of this research, more and more scholars put forward the concept of mixed payment: Luo et al. (12), Pan (13) and Wang (14) all think that a single payment method based on service items can't effectively control medical insurance fund fees for patients' medical expenses and hospital's economic benefits.

Based on these, the reform of payment method is related to the long-term operation of medical insurance fund and an important part of medical reform. At present, 11 provinces and cities in China have established comprehensive medical reform pilots, and actively promoted mixed payment methods such as per head, per disease, and total prepayment. Have the reform of medical insurance payment methods in these pilot areas achieved the purpose of cost control? Under this pilot background, this paper makes an empirical analysis on the effect of payment reform in the second newly-added pilot area, and explores the effect of medical insurance reform.

## Data and models

### Model selection

In this paper, the double difference method (DID) is used to explore the effect analysis before and after the medical insurance payment reform policy in China. “The basic idea of DID is that under a certain policy, the research objects are divided into treatment group and control group, the treatment group is the change before the policy influence, and the control group is the change result after the policy influence, but the reason for the change may involve the time factor, so it is introduced into the control group, because the control group will not be affected by the policy, and the change of the result before and after the policy of the control group is used to eliminate the time effect. Therefore, the double difference method reflects the policy effect based on the comparison of the differences before and after the policy.

Form method is used to reflect the idea of double difference: step one, calculate the mean values of group 1 [treatment group: E(Y_t1_), E(Y_t2_)] and group 2 [control group: E(Y_c1_), E(Y_c2_)] before and after the implementation of the policy. In the second step, the average value before and after the implementation of the policy in Group 1 is subtracted from the average value after the implementation of the policy in Group 1 to get the changes before and after the implementation of the policy in Group 2, and the same operation is performed in Group 2 to get the changes before and after the implementation of the policy in Group 2. Step 3: Subtract the change of Group 1 from the change of Group 2, and eliminate the time effect to get the policy effect. The process of two subtractions reflects the idea of double difference. The specific process is shown in Table 1.

**Table 1:** DID model.

	**Before change**	**After change**	**Difference**
Group1 (Treat)	E(Y_t1_)	E(Y_t2_)	ΔY_t_=E(Y_t2_)-E(Y_t1_)
Group2 (Control)	E(Y_c1_)	E(Y_c2_)	ΔY_c_=E(Y_c2_)-E(Y_c1_)
Difference			ΔΔY = ΔY_t_-ΔY_c_

The regression method is used to reflect the double difference model. Specifically, the model of the double difference method is as follows:


$$\begin{array}{l} Y_{it}=\beta_{0}+\beta_{1}treat_{i}+\beta_{2}P_{t}+\beta_{3}treat_{i}P_{t}+\varepsilon_{\text{it}} \end{array}$$

Among them, treat is a grouping dummy variable. If i is affected by the policy, it belongs to the processing group, and the corresponding treat value is 1. If i is not affected by the policy, it belongs to the control group, and the corresponding result value is 0, Pt is the dummy variable of the policy implementation, Pt is 0 before the policy implementation and Pt is 1 after the policy implementation. Treat i·Pt represents the interaction between virtual variables of different groups and virtual variables of policy implementation, and its β_3_ coefficient reflects the net effect of policy implementation. Once again, the table shows the β_3_ role. From the Table 2, we can see that β_3_ size and direction of the table reflect the dual differential policy effect, while the size and direction of the table reflect the time effect.

**Table 2:** β_3_ Effects in the DID model.

	**Before change**	**After change**	**Difference**
Group1 (Treat)	β_0_+β1	β_0_+β1+β_2_+β3	Δ*Yt* = β_2_+β3
Group2 (Control)	β_0_	β_0_+β2	Δ*Y*_*c*_ = β_2_
Difference			ΔΔ = β_3_

### Data selection

This paper selects the annual data of 27 provinces in five periods from 2013 to 2017, including 7 newly added reform pilot provinces in 2016, sixteenprovinces that did not participate in the reform pilot in 2016 and four provinces that carried out comprehensive medical reform pilot in 2015. The data of all provinces are from China Health Statistics Yearbook and China Statistical Yearbook.

To study the control effect of medical insurance payment reform on medical insurance expenses, this paper takes “the current balance of employee medical insurance fund” and “the growth rate of employee medical insurance fund expenditure” as independent outcome variables to evaluate the implementation effect of medical insurance payment reform. Among them, the data of the explained variable “employee medical insurance fund current balance” is obtained by subtracting “current fund income” from “current fund expenditure,” and “employee medical insurance fund expenditure growth rate” is the year-on-year growth rate compared with last year. Whether to implement the medical insurance payment reform policy is the key explanatory variable of this study. In this paper, 11 pilot provinces are set as “treatment group” and 16 non-pilot provinces as “control group”. The data from 2013 to 2015 are before the implementation of the policy and the data from 2016 to 2017 are after the implementation of the policy. At the same time, this paper selects some control variables related to medical insurance fund expenditure. Rui and Benfeng (15) analyzed the influencing factors of basic medical insurance fund expenditure, and found that medical and health expenditure, utilization of health services, supply of health services, number of medical insurance participants and other factors may affect medical insurance fund expenditure. Therefore, in this paper, the number of employees participating in medical insurance, the number of retirees, the total health expenditure, the per capita health expenditure, the number of public hospitals, the number of outpatients in public hospitals and the number of inpatients in public hospitals are analyzed. STATA13.0 software is used for data processing.

Table 3 is descriptive statistics of the mean values of main variables in the treatment group and the control group, reflecting the changes of basic data before and after the implementation of the policy. The current balance of the two groups of basic medical insurance funds for urban workers has increased after the pilot, and it is difficult to judge which group has the greater increase only from the comparison of average value. From the average change of the growth rate of the two groups' fund expenditure, the decrease of the growth rate of the control group after the pilot is greater than that of the treatment group. Among the control variables, the total health expenditure and the proportion of total health expenditure to GDP in the two groups also increased after the pilot, and the increase range of the control group was significantly larger than that of the treatment group. The per capita health expenditure is opposite between the treatment group and the control group. The average value of the treatment group increased after the pilot, while that of the control group decreased significantly after the pilot. The number of public hospitals in both groups decreased after the pilot, while the number of outpatients and inpatients in public hospitals increased after the pilot. From the point of view of insurance coverage, the number of on-the-job and retired people has increased, the influence of medical insurance on residents has been deepening, and the number of insured people may increase with the change of time. Therefore, from the comparison of the mean value between the treatment group and the control group, the mean value of both groups has increased after the pilot.

**Table 3:** Descriptive statistics of main variables.

**Variables**	**Treatment group**		**Control group**	
	**Before the pilot**	**After the pilot**	**Before the pilot**	**After the pilot**
Current balance of the basic medical insurance for urban employee	74.25	109.17	32.45	38.27
Expenditure growth rate of the basic medical insurance for urban employee fund	0.19	0.15	0.15	0.10
Total health expenditure	1,273.21	1,740.32	1,119.11	1,526.57
Per capita health expenditure	3,238.83	4,191.72	6,420.26	3,723.36
Number of public hospitals	396.19	373.86	459.15	432.58
Number of outpatients in public hospitals	9,631.85	10,710	7,912.24	8,845.76
Number of inpatients in public hospitals	446.14	518.81	419.12	483.87
Number of employees participating in the Basic Medical Insurance for Urban Employee	697.48	745.43	656.40	681.30
Number of retirement employees participating in the basic medical insurance for urban employee	261.43	288.07	222.73	241.31

## Empirical result analysis

### DID estimation result

From the results of double difference estimation (as shown in Table 4), the first column and the second column are cases where the control variables are not increased or increased, respectively. The DID regression results show that whether the control variables are added or not, the reform of payment methods has a significant impact on the current balance of employee medical insurance fund. From the regression results of the current balance of medical insurance fund, the difference between the control group and the experimental group before and after the policy is 0.48, and the difference between the two groups is 1.04, both of which are statistically significant. However, from the regression results of the growth rate of medical insurance fund, there is no significant correlation and lack of statistical significance. Compared with the control group, the current balance of regional medical insurance funds with the reform of payment method increased by 56.2%, and passed the test at a significant level of 5%.

**Table 4:** DID result.

	**Current balance of the basic medical insurance for urban employee**	**Expenditure growth rate of the basic medical insurance for urban employee fund**	**Current balance of the basic medical insurance for urban employee (control)**	**Expenditure growth rate of the basic medical insurance for urban employee fund (control)**
Before diff	0.487** (18.852)	0.039*** (0.018)	0.105** (13.604)	0.049*** (0.018)
After diff	1.049*** (23.089)	0.043** (0.018)	0.547*** (16.570)	0.054** (0.022)
Diff-in-Diff	0.562*** (29.080)	0.004 (0.025)	0.242*** (20.925)	0.004 (0.027)
Total health expenditure			0.048 (0.021)	0.025*** (0.001)
Per capita health expenditure			0.000 (0.000)	0.013*** (0.000)
Number of public hospitals			0.014 (0.041)	0.000*** (0.007)
Number of outpatients in public hospitals			0.015*** (0.002)	0.001 (0.004)
Number of inpatients in public hospitals			-0.241*** (0.049)	0.011*** (0.019)
Number of employees participating in the basic medical insurance for urban employee			−0.088*** (0.027)	0.000*** (0.001)
Number of retirement employees participating in the basic medical insurance for urban employee			0.171*** (0.049)	0.000*** (0.000)

**Means significant correlation at the level of 95%, ***means significant correlation at the level of 99%.

Control variables are introduced into the model to control other factors that may have an impact on medical insurance expenses, including total health expenses, per capita health expenses, the number of public hospitals and outpatient clinics, the number of inpatients, and the number of medical insurance employees and retirees in urban areas. Compared with not adding control variables, the current balance of medical insurance fund increases slightly, but the implementation of medical insurance payment reform policy can still significantly improve the current balance of medical insurance fund. By introducing control variables, it is proved once again that the reform of payment mode has no significant effect on the growth rate of medical insurance fund expenditure. The reason may be that the growth rate of medical insurance fund expenditure is influenced and restricted by other factors. It can be seen that after the comprehensive medical reform pilot in 2016, compared with the provinces that did not participate in the pilot, the current balance of the employee medical insurance fund in the 11 participating pilot provinces increased significantly, further indicating that the implementation of the medical insurance mixed payment method has a significant effect on the control of medical insurance expenses.

### Applicability test of double difference model

#### Balance test

The condition of balance requires that there is no systematic difference between two groups of explanatory variables, so according to the *T-*test of differences between two groups, this paper checks whether there is any difference between each variable and two groups (see Table 5). By comparing the results, we can see that there are differences between the two groups. This shows that the choice of explained variables is appropriate and effective. The *P*-values of other control variables are all above 0.05, which indicates that there is no significant difference in the average effect of control variables, so the distribution of two groups of explanatory variables is consistent.

**Table 5:** Balance test.

**Variables**	**diff**	**t**	**p**
Current balance of the basic medical insurance for urban employee	4.869	3.40	0.001***
Total health expenditure	154.10	0.88	0.379
Per capita health expenditure	−3.2e+03	0.52	0.606
Number of public hospitals	−62.96	1.07	0.288
Number of outpatients in public hospitals	1,719.61	1.01	0.315
Number of inpatients in public hospitals	26.93	0.39	0.694
Number of employees participating in the basic medical insurance for urban employee	41.076	0.25	0.800
Number of retirement employees participating in the basic medical insurance for urban employee	38.695	1.11	0.271

***Means significant correlation at the level of 99%.

#### Common trend test

**Figure 1:**
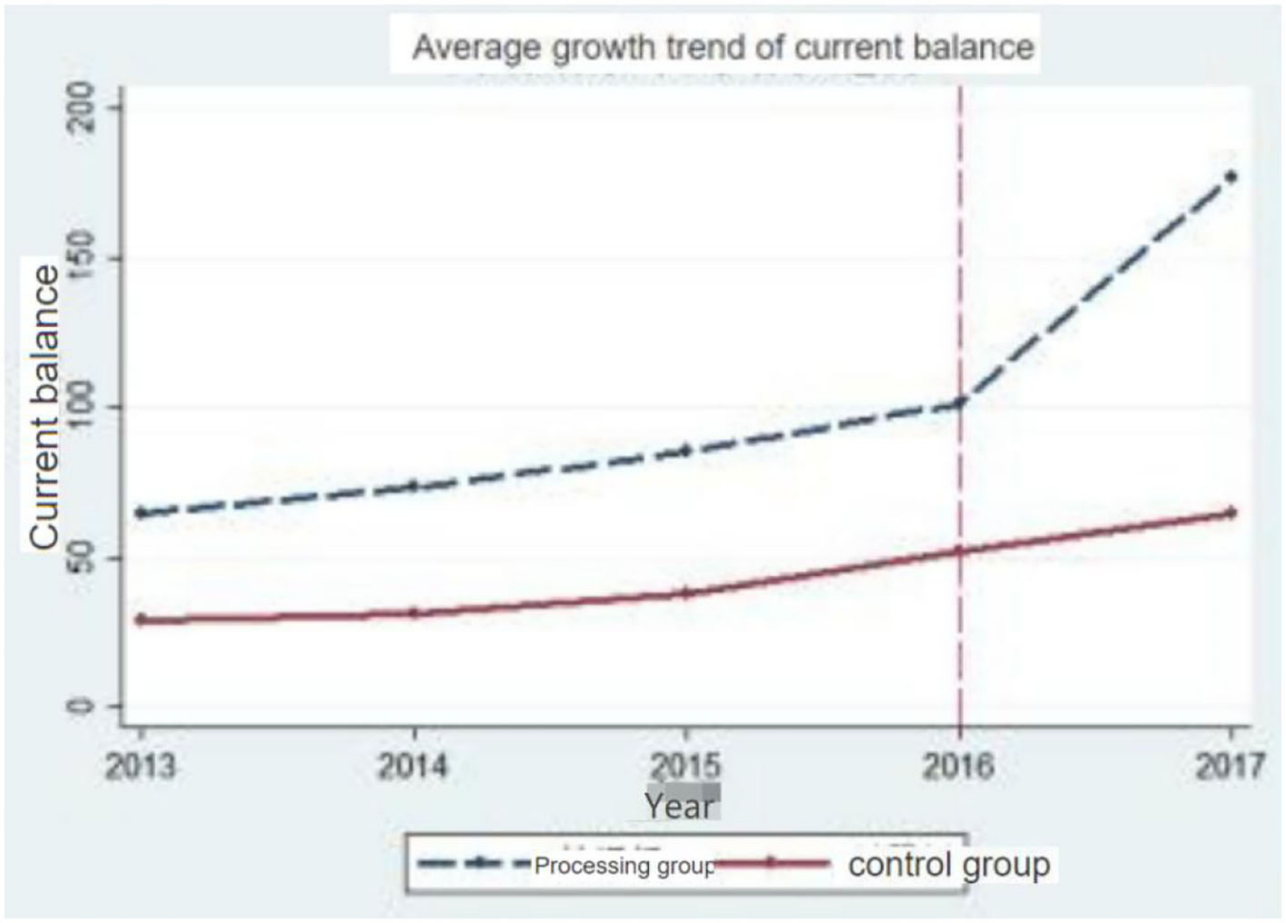
Parallel trends of current balances of medical insurance funds.

The common trend is very necessary for the double-subordination method, which indicates that the first group and the second group have a common trend before the implementation of the policy. Therefore, in order to verify the appropriateness of the DID model in this paper, the current balance of medical insurance funds in the treatment group and the control group is tested by common trend, and the time node of policy change in this paper is in 2016. Figure 1 shows that before the implementation of the policy, the current balance of the medical insurance fund for urban employees in the pilot provinces and those in the non-pilot provinces maintained the same growth trend. After the implementation of the policy, compared with the control group, the medical insurance fund balance of the treatment group increased significantly. Therefore, this paper uses DID model to test the influence of the reform of medical insurance payment mode on medical insurance expenses, which is a hypothetical condition in line with the common trend.

## Conclusions and suggestions

### Conclusion

Based on the policy of medical and health system reform, this paper studies the influence of mixed payment mode reform on medical insurance fund, establishes a double difference model, and analyzes the effect of the reformed payment mode on controlling medical insurance fund through the changes of main variables of two groups of data before and after the implementation of medical reform policy.

(1) The reform policy of medical insurance payment method has achieved the effect of cost control, and the current balance of medical insurance funds in pilot areas has increased slightly.

After the implementation of total amount control and various comprehensive payment mode reforms in the pilot areas, the current balance of the basic medical insurance fund for urban employees has increased significantly, but the pilot has no significant impact on the growth rate of medical insurance fund expenditure. After controlling the medical and health expenditure, the utilization of health services, the number of medical insurance participants and other factors, the growth rate of the fund's current balance decreased but remained significant. It can be seen that the effect of this pilot reform of medical insurance payment mode is in line with the policy objectives, and the effect of cost control has been achieved to a certain extent. The current balance of medical insurance funds participating in the pilot areas has increased slightly. However, the effect of this reform pilot on curbing the excessive growth of medical insurance fund is not ideal. Although this reform has controlled the growth rate of medical insurance fund balance to a certain extent, it has not effectively reduced the growth rate of medical insurance fund expenditure, and the policy of reducing medical insurance fund in terms of payment methods needs to be further improved.

(2) The reform of payment methods is influenced by many factors, and the control of medical reform fees still needs to be effectively improved.

Judging from the reform measures of medical insurance payment methods in seven new pilot provinces of medical reform in 2016, the reform path of each province is mainly to implement total budget and explore mixed payment methods. However, the medical level is different among provinces, the conditions for implementing various payment methods are different, and the medical insurance supervision methods are also different. Differences in medical level, conditions and management in different regions will potentially affect the implementation effect of the reform, and then affect the evaluation of the reform of payment methods. In addition, under the background of the double interweaving of the rapid development of medical technology and the aging of population in China, it is an important task of medical reform in today's era to effectively restrain the excessive growth of medical expenses. Therefore, although the pilot reform of medical insurance payment method in 2016 did not well-control the growth of medical insurance expenses, we chose payment methods according to the characteristics of different types of medical services, so as to control medical expenses to the greatest extent.

### Countermeasures and suggestions

(1) Define the reform direction of payment methods and implement mixed payment methods.

Before the reform of medical insurance payment method, payment by project was used in most parts of the country, and it was relatively extensive in management. In the areas that pay by items, hospitals often provide patients with many unnecessary examination items for their own benefit, which on the one hand leads to the waste of medical resources and on the other hand leads to a substantial increase in medical expenses. In 2011, 2015 and 2017, China proposed to implement total amount control, gradually reduce payment by project, and explore payment by project and per head. In areas where China implemented mixed payment methods, due to the change of the disadvantages of the original payment by project, some unreasonable waste of medical resources was reduced, and the purpose of controlling medical expenses was achieved. After many reforms, the payment policy has played a guiding role in controlling the behavior of medical institutions to a certain extent, but the phenomenon of over-examination and over-treatment in medical service institutions has not been effectively solved (10). Therefore, China should make clear the reform direction of payment methods, and achieve the purpose of controlling medical insurance fund expenses on the basis of implementing mixed payment.

(2) Improve the multi-component payment framework.

There are many types of medical services, and the demands of outpatient service, hospitalization service, primary medical service and long-term hospitalization service are complex. Single payment method cannot effectively adjust the allocation of medical resources and ensure the medical quality. China continues to promote the mixed payment method combining the post-payment system and the prepayment system, and some areas such as Shanghai and Zhenjiang have initially formed a multi-component payment pattern (11). However, there is a big gap between different regions. As the reform of payment methods generally adopts the pilot mode, the cities included in the pilot range have established mixed payment methods that are suitable for medical resources and management level according to local conditions. We should comprehensively promote the reform of mixed payment methods, drive the reform of other regions with the pilot areas, and gradually adjust according to regional differences, so as to form a nationwide multi-component payment mechanism.

(3) Establish and improve the payment reform system.

At present, the medical insurance monitoring system in most areas is not perfect, and the performance appraisal system of medical institutions and medical insurance agencies does not match. Therefore, medical insurance payment should improve the management and implementation of relevant policies from the institutional level. Strengthen coordination and communication among departments, establish a unified medical insurance monitoring index system, and focus on monitoring and evaluation of indicators such as total payment, person-times and average hospitalization expenses (16). Accelerate the construction of information system, build medical insurance cost monitoring system and medical insurance payment management system based on regional medical information system, combine daily settlement of medical insurance cost with intelligent monitoring, monitor medical expenses in real time, and strengthen supervision (17). Promote the construction of provincial-level medical insurance big data information system, conduct early warning and audit on all kinds of data, and strengthen supervision before and after the event.

## Data availability statement

Publicly available datasets were analyzed in this study. This data can be found at: http://www.stats.gov.cn/.

## Author contributions

JL put forward the research ideas and framework of this paper. JL and SW wrote the full text. LD data processing and analysis. RZ and LB combed the literature and checked and revised the full text. All authors have read and agreed to the published version of the manuscript.
